# Challenges faced by migrant populations in complying with public health measures during the COVID-19 pandemic in Malaysia: A qualitative study

**DOI:** 10.1136/bmjph-2024-000923

**Published:** 2024-09-04

**Authors:** Tharani Loganathan, Amirah Zafirah Zaini, Watinee Kunpeuk, Rapeepong Suphanchaimat, Huso Yi, Aysha Farwin, Hazreen Abdul Majid

**Affiliations:** 1 Centre for Epidemiology and Evidence-Based Practice, Department of Social and Preventive Medicine, Faculty of Medicine, University of Malaya, Kuala Lumpur, Malaysia; 2 International Health Policy Program, Ministry of Public Health, Nonthaburi, Thailand; 3 Division of Epidemiology, Department of Disease Control, Ministry of Public Health, Nonthaburi, Thailand; 4 Saw Swee Hock School of Public Health, National University of Singapore, Singapore; 5 School of Health and Rehabilitation Sciences, Health Sciences University, Bournemouth, UK; 6 Centre for Population Health, Department of Social and Preventive Medicine, Faculty of Medicine, University of Malaya, Kuala Lumpur, Malaysia

**Keywords:** COVID-19, Public Health, Public Health Practice

## Abstract

**Introduction:**

The COVID-19 pandemic adversely impacted migrants in Malaysia, raising concerns about the effectiveness of public health measures. This study aims to investigate challenges faced by migrant populations in complying with public health measures during the pandemic.

**Methods:**

We conducted 29 in-depth interviews with stakeholders between April 2022 and February 2023. Thematic analysis was conducted, and results were organised by major COVID-19 public health measures: (1) movement restrictions, (2) non-pharmaceutical interventions, (3) COVID-19 screening and testing and (4) quarantine, isolation and hospitalisations.

**Results:**

Migrants encountered difficulties complying with the movement control orders due to livelihood crises and a lack of understanding of regulations. Financial constraints hindered migrants’ ability to purchase quality face masks, and they lacked the comprehension of the importance of non-pharmaceutical interventions for disease prevention. In the absence of government intervention, non-governmental organisations and international organisations played an important role in providing essential food aid, health information, face masks and hygiene products, and other services to migrants. Despite encouragement to seek testing and treatment, migrants were deterred by fear of immigration enforcement and unaffordable fees. Overcrowded living conditions made physical distancing, isolation and quarantine challenging. Many avoided government-designated quarantine centres due to financial constraints and fear of arrest. Delayed medical treatment may have resulted in high COVID-19 mortality among migrants.

**Conclusions:**

The COVID-19 pandemic highlighted significant health disparities experienced by migrants in Malaysia, including the double health and livelihood crises, and limited access to essential health information, resources, healthcare and social protection. Urgent reforms are needed to ensure migrant-inclusive health policies, enhance outbreak preparedness and prevent unnecessary suffering and deaths among migrants during both pandemic and non-pandemic periods.

**WHAT IS ALREADY KNOWN ON THIS TOPIC**

- Although national health policies were considered inclusive during the COVID-19 pandemic, the magnitude of cases involving migrants in Malaysia raised concerns about the acceptability of public health measures for migrants.

**WHAT THIS STUDY ADDS**

- Malaysia’s exclusion of migrant populations from public health policy exacerbated their health and socioeconomic disparities. Despite efforts by non-governmental and international organisations to provide food aid, health information, hygiene products and other services, their initiatives proved inadequate given the scale of the problem.

**HOW THIS STUDY MIGHT AFFECT RESEARCH, PRACTICE OR POLICY**

- Serious reforms are necessary to establish inclusive public health policies that cater to the needs of marginalised populations and address their social determinants of health, both during pandemics and beyond. Key measures include incorporating migrants into universal health coverage schemes regardless of documentation status, implementing culturally sensitive, language-appropriate risk communication strategies, combating xenophobia and discrimination in all policies, and fostering consistent whole-of-government approaches supported by multisectoral collaboration.

## Introduction

 The COVID-19 pandemic has had devastating health and socioeconomic impacts on migrant populations, who often live and work in crowded environments, face barriers to accessing healthcare and experience precarious employment.^1^,^5^

Malaysia, a major migrant destination country, hosted approximately 10% of its population as non-citizens in 2023.^6^ Among them, around 2.7 million were the low-skilled and semiskilled migrant workers from 15 countries, with the majority from Indonesia, Bangladesh and Nepal.^7 8^ However, the actual number of migrant workers was potentially as high as 5.5 million, including undocumented workers.^9^,^11^ Malaysia also hosts forcibly displaced migrants, with 182 010 refugees and asylum-seekers registered with the United Nations High Commisioner for Refugees (UNHCR) as of July 2023. The majority of these individuals are Rohingya from Myanmar, but Malaysia receives refugees and asylum-seekers from a total of 50 countries.^12^

Despite its status as a significant destination for migrants, Malaysia has not ratified international agreements safeguarding the rights of migrant workers or acknowledging the status of refugees.^13^,^15^ Also, immigration law fails to differentiate between refugees, asylum-seekers and undocumented migrants, leading to restricted access to healthcare, social protection and formal employment for all these groups, with risks of arrest and detention.^16^,^22^

Malaysia adopted a whole-of-government approach to respond to the COVID-19 pandemic, establishing a multiagency, centralised coordination council. The Ministry of Health (MOH) Malaysia spearheaded the national health response,^23 24^ implementing key strategies including movement restrictions and non-pharmaceutical interventions (NPIs), conducting targeted screening and testing, establishing institutionalised isolation and quarantine measures and facilitating hospitalisation for COVID-19 cases.^24^ Under the Prevention and Control of Infectious Disease Act 1988 (Act 342), a nationwide stay-at-home home order known as the Movement Control Orders (MCOs) was enforced.^25 26^ Alongside movement restrictions, NPIs such as mask-wearing, hand hygiene and physical distancing were mandated, with penalties of up to RM1000 (US$214) or up to 6 months in jail, or both, for non-compliance with regulations.^27 28^

Even before the pandemic, migrants faced significant barriers to accessing healthcare, predominantly stemming from financial constraints, absence of identity documentation and communication challenges.^19^,^22^ Moreover, they were more susceptible to deadly infectious diseases.^29^,^32^ During the COVID-19 pandemic, the MOH Malaysia reported that 58% of the 6774 recorded COVID-19 clusters as of 26 February 2022 were workplace related, with migrants comprising 63% of cases.^33^ Alarmingly, approximately one in five COVID-19 deaths in Malaysia were brought in dead (BID),^34^ with non-citizens facing a fourfold higher likelihood of being BID compared with citizens, underscoring concerns regarding the effectiveness of pandemic public health measures and healthcare accessibility for non-citizens.^35 36^

While national health policies were generally perceived as inclusive, the acceptability of these policies has been questioned due to the significant involvement of migrants in COVID-19 cases in Malaysia. This study aims to explore perspectives from multiple stakeholders to better understand the challenges encountered by migrant populations in adhering to public health measures during the COVID-19 pandemic.

## Materials and methods

This study is part of a larger project evaluating policy responses to the COVID-19 pandemic for low-income migrant populations in Malaysia, Thailand and Singapore. Here, ‘migrant populations’ refer to documented and undocumented low-income migrant workers, refugees and asylum-seekers in Malaysia. Notably, international travellers, students and expatriates were not included in this study. (Refer online supplemental appendix 1 for the definition of terms)

### Participant recruitment

Key informants with expertise in migrant communities’ issues during the pandemic in Malaysia were purposively sampled. This included individuals from non-governmental organisations (NGOs), international organisations, labour unions, policy stakeholders, healthcare providers and migrant communities. Participants were identified through an initial desk review and the research team’s knowledge of key actors in migrant health, leading to the creation of a database based on professional background and experience with Malaysia’s migrant communities. Invitations for potential participants were extended via telephone and email, with additional participants identified through snowball sampling. Recruitment continued until saturation was achieved, and no new information was anticipated.

### Data collection

**Figure 1:**
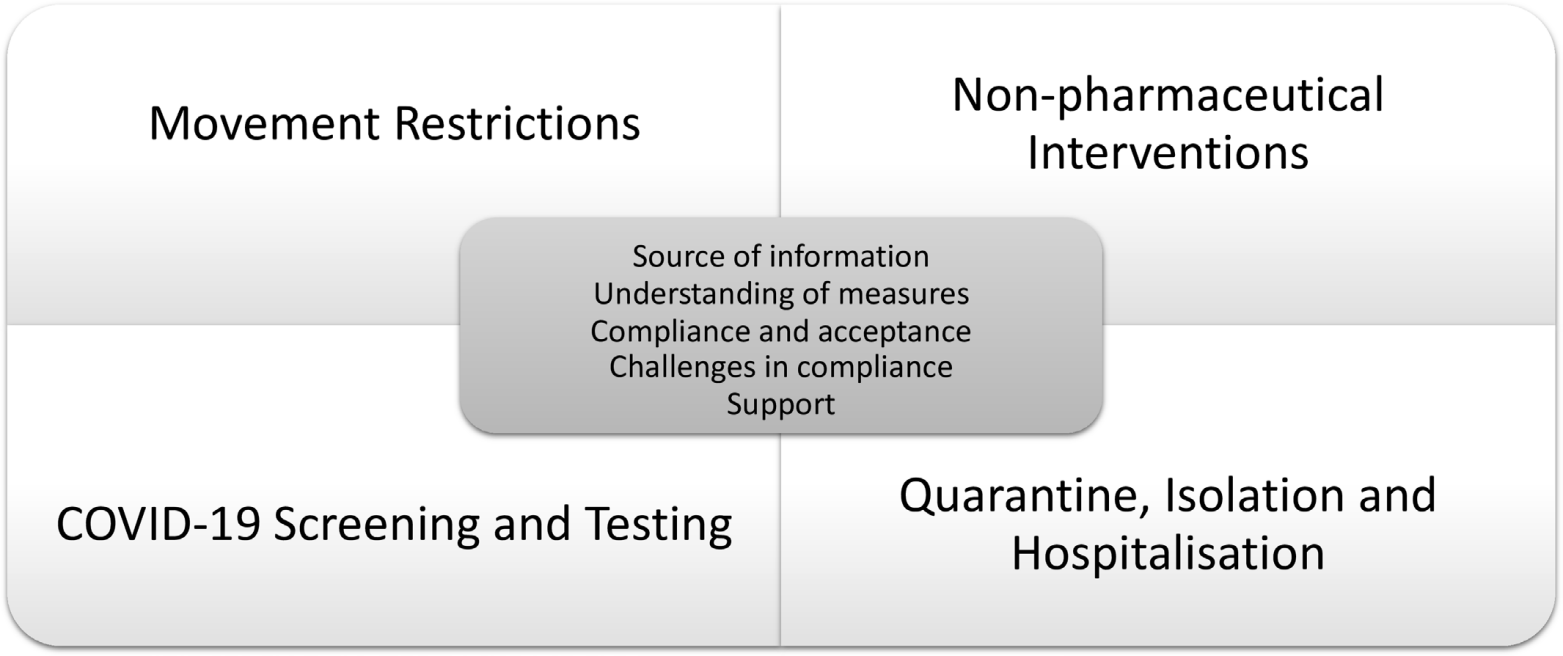
Framework for evaluating migrant populations’ response to public health measures during the COVID-19 pandemic in Malaysia.

Semistructured interview guides were developed following a desk review examining containment and closure, economic support, and health policies during Malaysia’s COVID-19 pandemic from January 2020 to February 2022. The guides contained introductory questions to ascertain participants’ backgrounds, followed by open questions on movement restrictions, NPIs, COVID-19 screening and testing, and quarantine, isolation, and hospitalisation. Key questions aimed to assess responses to public health measures focusing on the source of information, understanding of measures, compliance and acceptance, challenges in compliance and support (see figure 1).

Interview guides were customised to the background of the interviewees, with separate guides developed for three groups: (a) migrants (including migrant workers, refugees and asylum-seekers), (b) key stakeholders (comprising NGOs, labour unions, academia, government officials, etc) and (c) healthcare workers (representing the public sector, private sector and medical NGOs). See online supplemental appendix 2 for interview guides.

From 6 April 2022 to 20 February 2023, a total of 29 in-depth interviews were conducted, involving 32 individuals. While most interviews were conducted individually, three interviews involved two participants from the same organisation.

Participants comprised representatives from seven NGOs, five international organisations, two labour union activists, two policy stakeholders and six healthcare providers. Additionally, 10 migrant community representatives were interviewed including 6 representing migrant workers originating from Indonesia, the Philippines, Nepal and Bangladesh, and 4 representing refugee and asylum-seekers from Myanmar, Iraq and Pakistan. Table 1 provides an overview of study participants’ characteristics.

**Table 1:** Characteristics of the study participants (n=32)

Participant background	Label	No.
Non-governmental organisations	NGO	7
International organisations*	IO	5
Labour unions	LU	2
Policy stakeholders†	POL	2
Healthcare providers	HP	6
Migrant community representatives		
Migrant worker	MW	6
Refugee and Asylum-Seeker	REF	4
Total		32

* Of the five representatives of international organisations interviewed, three were also healthcare providers.

† The two policy stakeholders interviewed were also healthcare providers.

Interviews were conducted by researchers (AZZ, HAM and TL) in either English or Malay, based on participants’ preferences. Field notes and impressions were documented for each interview. 11 interviews were conducted face to face while 18 interviews were conducted online. On average, interviews lasted 50 min. Audio recordings were transcribed verbatim. Concurrent data analysis was conducted to guide data collection and refine interview guides.

### Data analysis

Data analysis employed immersive, exploratory and inductive methods, with regular discussions to refine codes and identify themes, following Braun and Clarke’s thematic analysis framework.^37^ Transcripts were edited for accuracy, coded into themes using NVivo V.12 Pro software and reviewed alongside field notes and impressions to refine codes and themes. Ongoing discussions were held to further refine codes, address negative cases and minor themes. Interviews conducted in Malay were analysed in the same language, with extracted quotations translated as needed.

### Reflexivity

Interviews were conducted by academic researchers from a reputable public university, who could be considered as trusted authority figures. To counter possible power imbalances, particularly among vulnerable migrant populations, participants chose interview times and locations.

### Patient and public involvement

Patients and the public were not directly engaged in the design and conduct of this study. However, study findings will be shared with the public and relevant communities through presentations and distribution of policy briefs, reports and publications.

## Results

Results were organised by the major COVID-19 public health measures in Malaysia, including (1) movement restrictions, (2) NPIs, (3) COVID-19 screening and testing, (4) quarantine, isolation and hospitalisations. Box 1 summarises the main study findings.

Box 1 **Main findings of the study**

**Movement restrictions**

- Difficulty complying with movement restrictions due to livelihood crises.
- Limited understanding of movement restrictions due to inadequate communication.
- Authorities’ treatment results in a loss of trust in the Malaysian government.

**Non-pharamaceutical interventions**

- Lack of financial means resulted in the reusing of masks and use of poor-quality masks.
- Difficulty adhering to physical distancing due to overcrowded housing environments.
- Lack of understanding of the importance of practising non-pharmaceutical interventions.

**COVID-19 screening and testing**

- Migrants avoided COVID-19 screening and testing for fear of arrest.
- COVID-19 tests were unaffordable, and migrants would have to pay for themselves.
- Inadequate knowledge of the COVID-19 testing availability and the importance of tests.
- Negative COVID-19 test requirements for return to work pushed migrants to get tested.

**Quarantine, isolation and hospitalisation**

- Home quarantine was impossible for migrant workers and refugees.
- Undocumented migrants avoid quarantine at government-designated centres for fear of arrest.
- Revocation of free quarantine for non-citizens at government-designated centres.
- Delay or avoidance of hospitalisation may explain increased deaths among migrants.

### Movement restrictions

#### Difficulty complying due to livelihood crisis

Movement restrictions and stop-work orders implemented during the MCOs were key measures taken by the Malaysian government to curb the spread of COVID-19. However, community representatives noted that despite the government’s requirement for employers to continue paying workers, many migrants were unpaid or received only partial salaries due to lax enforcement of labour laws. Consequently, many migrants were summarily dismissed and struggled to find new employment as businesses closed.

With closed international borders, migrant workers remaining in Malaysia experienced job losses, food shortages and evictions, making compliance with movement restrictions challenging. Participants suggested that migrants found violating stay-at-home orders may have been compelled to seek work to support themselves or their families.

Migrant communities, commonly engaged in low-wage employment, typically have limited financial reserves to endure crises. Particularly vulnerable were undocumented migrant workers and refugees lacking legal employment rights in Malaysia. Interviewees recounted instances of refugees being arrested for non-compliance of MCOs, as they sought work or income opportunities for survival.

Even from before the pandemic, my community members live from day-to-day [they have no savings]. The husband earns money on that day when he works, and [only] then he gets to eat [and the family gets to eat]. Because the husband goes to work in the morning and may only earn 20 to 30 [ringgit], and he buys food in the evening and cooks at night. When the lockdown suddenly happened, many people didn’t even know that the lockdown had happened. The Rohingya is a normal person, he still goes out and collects iron [to sell]. But suddenly people beat him. Many Rohingya people were arrested for violating the MCO. REF-003 (Translated from the Malay language)

Interviewees reported minimal assistance from employers and the government for migrant communities during the lockdown. Instead, NGOs, international organisations and the Malaysian public rallied to provide food aid to marginalised communities, particularly during the initial months of lockdown.

#### Limited understanding of movement restrictions

##### Information distributed was not in migrant languages

Information regarding the COVID-19 crisis and MCO regulations was officially disseminated by the government in Malay and English languages, which may not be understood by migrants. Even Indonesian workers and others fluent in informal Malay might struggle with the formal Malay or English used in official messaging. Study participants emphasised the importance of sharing MCO regulations, as they were legally enforced, with migrants facing arrest or fines due to lack of awareness or understanding.

Illiterate individuals, particularly from the Rohingya refugee community, faced additional challenges. Study participants explained that NGOs and international organisations in collaboration with migrant community leaders, developed video and audio recordings of health information for dissemination through social media and messaging platforms.

… there are certain communities, for example, the Myanmar community… They cannot speak our language. If you understand about the refugee community, [like] the Myanmar [communities], they have so many tribes there like the Chin, Kachin, and other ethnic groups. So, we certainly like showing them video clips and showing them on YouTube, [that] is a good example of how we reach out to them. Because a picture explains a million words. NGO-001

Some organisations established hotlines to provide migrants with a reliable source of information, eventually leveraging these platforms to organise food aid for those facing livelihood crises.

Additionally, migrant-led organisations created grassroots translations of MCO-related messages for dissemination within their communities while international organisations provided standardised translated health messaging vetted by experts. However, participants noted challenges in promptly translating information due to rapidly changing government regulations and difficulties in accurately translating to multiple migrant languages.

Ensuring information reached migrants was also challenging, as not all had access to digital platforms due to reasons such as lack of smartphones, digital illiteracy or poor internet connections in rural areas, limiting communication and information dissemination during the pandemic.

##### Difficulty with the MySejahtera App

The MySejahtera mobile app played a crucial role during the pandemic by disseminating official COVID-19 health information and indicating the bearer’s COVID-19 status and risk level. Different risk statuses required varying measures such as quarantine or self-isolation. Additionally, prior to entry into any premise, the premise’s QR code must be scanned, and the individual’s risk status is displayed on the MySejahtera app. Only those with a green or yellow colour display indicating low-risk status would be allowed entry.

NGOs reported that migrants struggled to understand risk statuses and comply with movement restrictions due to the app’s confusing display and lack of information in migrant languages.

We did hear of the breach of the movement restrictions for the patients which were also significantly related to the lack of understanding of the MySejahtera application, and [what] it actually meant. And you remember, we had the colour system in place [colours representing the risk status in the MySejahtera apps]. And that is also quite difficult even to explain [to migrants] that you have the different shades of Orange [in the risk status] and one of the Orange [status] means—YES, you are OKAY. And the other shade of the Orange means like, actually—NO, you should not go anywhere. And even for us, it was very difficult to explain to them. IO-003

Additionally, the high reliance on the app posed challenges for migrants, as not everyone owned a smartphone. Illiterate Rohingya refugee women were particularly vulnerable, often sharing a smartphone among family members, and thus unable to move freely.

### Authorities’ treatment of migrant communities

#### Lack of communication from authorities on lockdown measures

The enforcement of Enhanced Movement Control Order (EMCO) in migrant residential areas identified as COVID-19 hotspots was often abrupt, with little warning provided to residents. These areas were cordoned off by the police and army with barbed wire, restricting entry and exit while residents faced stay-at-home orders, business closures and limited food supply during the minimum 2-week lockdown. Participants noted that migrants did not receive adequate explanation or support from the government during this challenging period, making cooperation difficult.

So, when suddenly there was a big outbreak, suddenly the army barricade came in [EMCO enforced]. They [migrants] cannot move at all. So, during that time, there was a lack of communication between the MOH and the people [migrants], to fully understand the reason that they did the barricade. NGO-001

Participants highlighted the harsh conditions endured by non-citizens during EMCO lockdowns, as government food support were exclusively allocated to citizens. Non-citizens relied on food aid from NGOs; however, delivering aid to migrants in EMCO areas posed challenges as NGOs were prohibited from entering these restricted locations.

#### EMCO was followed by immigration raids

Before the pandemic, migrants distrusted healthcare personnel due to immigration status checks at government health facilities. Despite reassurances by the MOH Malaysia that undocumented migrants would not face immigration action, this participant noted a shift in trust occurring after 1 May 2020 when immigration raids and arrests occurred following lifting of EMCO measures in residential apartments in Masjid India, Kuala Lumpur.

… we told them [the government] that ‘Well, nobody is going to come forward unless you say you're not going to arrest them’. Because for years, anybody without documents [if they] go to hospitals, you arrest them. So, you don't expect one night, they are going to trust you. [And] in a time like this, when you're calling out for them to come, all the more they’ll be so fearful. So, a lot of NGOs together with the UN [the United Nations organisations based in Malaysia], sent a letter to the Prime Minister, requesting that they make an announcement for no arrests. On March 22nd or 23rd, that announcement finally came and said ‘No arrest. Whoever you are, no arrest. Come forward for your screening.’ It was good. Up to May 1^st^ [2020] when the EMCO in Masjid India was lifted, and that was when they went in and arrested all the people in Masjid India. So, that marked the point of breaking of trust. Because up to then, people happily went for screening and were not worried. After that, they would not even come out for food. IO-002

Immigration raids and detentions of undocumented migrants following public health lockdowns eroded trust in the Malaysian government among non-citizens, leading to evasion of authorities and resistance to public health initiatives, such as COVID-19 screening, quarantine and vaccinations.

### Non-pharmaceutical interventions

#### Lack of financial resources to purchase masks

Under Act 342, an RM1000 fine was imposed on individuals not wearing face masks in public spaces, including workplaces, with employers responsible for providing masks and other hygiene products for employees. However, participants observed that workers often had to purchase masks themselves and migrant workers would reuse masks to reduce costs while complying with the law, thereby increasing vulnerability to COVID-19.

[They were] complying [with the mask requirement], but migrant workers were wearing the cheap ones, the blue ones [single-use masks]. They have to wear it for two or three days. It is wrong, you know? The employers said, ‘Eh, [masks] are very expensive’. [At] that time, the price was high also. ‘It was too expensive, we cannot buy [workers]’. So, they were wearing it for days. Even though it was dirty, they were wearing it. [For them] they are just complying [with requirements]. ‘We are wearing a mask’. [But] whether it’s clean or not [it’s not their concern]. They were suffering. LU-001

NGOs and international organisations provided face masks and hygiene supplies alongside food aid to migrant communities during the MCOs. Additionally, refugee communities were observed making cloth face masks for personal use and selling them to raise funds.

#### Difficulty adhering to physical distancing due to overcrowded housing

Migrants faced challenges in practising physical distancing due to crowded living conditions, particularly evident among refugees who often share single-rented apartments among multiple families. Overcrowded housing for migrant workers was linked to employers’ non-compliance with the Workers’ Minimum Standards of Housing and Amenities (Amendment) Act 2019 (Act 446), coupled with inadequate enforcement. Such non-compliance contributed to numerous workplace COVID-19 outbreaks at migrant workers’ housing since November 2020.

COVID-19 brought forward two things. Which is [firstly] the necessity for Act 446. And number two, the truth of what had actually been happening. It just opened up that doorway. It showed us how bad their [migrant workers] living standards were. And if the living standards [Act 446] were complied with, this would not have happened. The migrant workers would not have had COVID, there would not have been these mass COVID clusters for migrant workers. If you look at all the clusters of migrant workers, it was always because of where they were staying, which is the responsibility of the employer… LU-002

Participants reported that workers’ accommodations were makeshift, poorly constructed, unhygienic and overcrowded, rendering physical distancing, quarantine of exposed persons and isolation of the sick impossible.

#### Lack of understanding of the importance of practising NPIs

Health information, even when translated into migrant-friendly languages, often contained confusing scientific jargon that hindered migrants’ comprehension of the importance of the mandated NPIs.

Even with translations [it] is not necessary [that] they get the full comprehension of what is being requested. So, you have to spend a bit more energy [and] time to explain to them, have a conversation [with them]. Just putting a poster or a voice recording [is not enough]—their culture is [that] normally, they don't ask to clarify. They will just look [and] process [the information] according to what would be convenient [for them]. Not necessarily, what is ‘right’ or what is ‘wrong’ or ‘illegal’ or ‘legal’. So, then you really have to spend triple the amount of energy to make sure they understand. So, imagine if an NGO like us [with] 10 to 20 years of experience, and we [still] struggle with communication issues. I don't know how general information will be helpful. I've met migrants who can't write and read. Some [even] cannot spell their names. So, how do you [expect them to] understand complex information like the word ‘symptomatic’? But nobody could understand it. Then you don't follow instructions because you don't understand. NGO-002

This lack of understanding led migrants to prioritise compliance with regulations to avoid penalties rather than personal protection. Consequently, they focused on wearing masks to avoid fines, neglecting aspects like mask quality, cleanliness, hand hygiene or physical distancing, which were less enforceable.

Actually, to be honest, they [migrants] do not believe in COVID-19. They do not believe [it]. So, they just wear the mask because of the place [masks were required in public]. But they do not use hand sanitisers and other [NPIs] things. They do not believe. They just think [that] this [COVID-19] is just a test come from Allah SAW. REF-001 (Translated from the Malay language)

### COVID-19 screening and testing

#### Avoiding COVID-19 testing for fear of arrest

Undocumented migrants, in particular, were reluctant to undergo COVID-19 testing due to fear of arrest. Participants noted intensified distrust and lack of cooperation with health authorities following immigration raids post-EMCO lockdowns in specific COVID-19 hotspot locations.

It is like when healthcare workers come to this area, and then set up tents, and conduct COVID-19 testing. After that, many people were arrested. Then, what the community understands, is that when healthcare workers come in, they will also arrest people [migrants]. REF-003 (Translated from the Malay language)

Despite only undocumented migrants being at risk of immigration arrest, all migrants experienced fear and mistrust due to widely publicised immigration arrests. Government policies and messaging may have inadvertently fueled xenophobic sentiments and diminished migrants’ willingness to seek testing and treatment.

When it comes to testing, there were all this scapegoating and negative sentiments toward migrants you know? All that fear-mongering that migrants were the source of this increased [COVID-19 case] numbers. And they became the victim of the hate and racism and all of that, based on government policies and also the rhetorics that were coming out at the time by the immigration [department] and others. So, that didn't help the situation. I mean, which migrant would want to come forward, if they are going to get deported? And so, predictably, they would go underground. In terms of public health, that was also predictable and a disaster. POL-001

Participants observed reluctance among symptomatic migrants to seek testing, raising concerns about ongoing COVID-19 transmission. However, easier access to private healthcare facilities and NGO-based services later in the pandemic facilitated testing without fear for migrants, who were more willing to receive care from trusted providers.

#### COVID-19 tests were expensive

In January 2020, MOH Malaysia waived fees for COVID-19 testing for symptomatic non-citizens suspected of infection, with positive cases exempted from fees for investigation, treatment and hospitalisation. However, participants noted that this free testing was limited to suspected cases testing positive while others faced expensive charges for testing, at non-citizen rates. The time-consuming nature of RT-PCR tests also deterred migrants from seeking testing at government clinics.

I would say [they would] hardly go for testing, because there’s no point to do so. For them [it is] like, ‘Why do I need to do? I need to fork up 300 ringgits to do what [screen for COVID-19]? I don't even have food. Because of my health? No!’. IO-001

In December 2020, the government mandated that employers cover the costs of COVID-19 testing for all employees, yet a labour union representative suggested that employers might not conduct workers testing or resort to salary deductions to cover testing costs for migrants.

Employers were supposed to pay. So, what happened was that some companies said that if you have symptoms, [or] if you face any issues, you [should] go [and] get it tested and pay, [and] keep the receipts [for claiming]. They [some employers] did that. [But the] majority did not. The majority [of employers] were like, ‘You are on your own.’ They didn’t even give [migrant workers’] food, so forget about paying for COVID tests. LU-002

Community activists highlighted that refugees and undocumented migrants, in particular, avoided seeking healthcare due to the fear of immigration arrests and their inability to afford expensive tests, rendering these groups particularly vulnerable.

Policy stakeholders interviewed suggested that the government’s preference for gold standard RT-PCR tests over the cheaper rapid test kit (RTK) may have restricted the availability of COVID-19 testing. Nevertheless, when these were available, certain private healthcare facilities and industries offered free RTK tests to migrant communities through trusted NGOs, which were well received. This assistance alleviated the financial strain of seeking timely health interventions and potentially contributed to preventing the spread of COVID-19.

#### Inadequate knowledge of the availability of COVID-19 screening and testing

Participants noted that migrants lacked the information needed to make informed decisions about seeking care for COVID-19, including awareness of testing services, locations, types of tests, and associated costs.

Earlier, where do we want to do the testing? A lot of people don't know. It was not like now that many clinics can do it. The test kit [RTK] didn't even exist before. MW-001

They were also unaware of the importance of testing for symptomatic individuals or those in close contact with COVID-19-positive individuals. This lack of knowledge and understanding likely contributed to delays in seeking healthcare and poorer outcomes.

#### Negative COVID-19 test requirements for returning to work pushed migrants to get tested

In January 2021, the government mandated a negative COVID-19 test result for returning to work, aiming to facilitate a safe reopening of the economy. Despite the costliness of the tests, this participant explained that the testing requirement created an economic imperative for migrants and their employers to obtain negative COVID-19 test results.

No. Most of them would not come forward for COVID screening until one time when [the] MCO was a bit loosened and some of their bosses already starting the business, and they only want those who have been tested negative [to return to work]. [So], to prove that they were negative and to work—because of work reason—economic reason, they were willing to be tested. That is the main reason, I would say of why they [finally] came forward to get tested. IO-005

### Quarantine, isolation and hospitalisation

#### Home quarantine was impossible for migrants

Isolation of infected individuals and quarantine of those exposed individuals were crucial public health measures employed to curb the spread of COVID-19 during the pandemic. Participants claimed that migrant workers, refugees and asylum-seekers were unable to adhere to isolation or quarantine due to their shared housing arrangements.

… for refugees and migrant workers was that social distancing was just not possible—right? I mean, they live in very small places, very small quarters with A LOT OF people. [For] migrant workers, it is a [place they share with a] lot of other workers. And with refugees, it’s very often with a lot of families squeezed into one flat. So just basically having the ability to even conduct that social distancing and kind of advice on, ‘Well, if you have COVID then isolate yourself in a room’. Like that. That just isn’t possible <laugh> You know what I mean. If you are in a place where literally you have a two-room flat, and each room has a family of six in it—then it is a shared space. Where can you isolate [yourself]? NGO-003

Most employers typically did not offer separate isolation facilities for COVID-19-infected individuals, potentially facilitating the spread of infection among workers’ accommodations. This participant highlighted that quarantining migrant workers became more feasible when centralised government-designated quarantine centres were opened to non-citizens.

But when one person gets COVID, everyone gets infected. That’s just how it is. The employer says, ‘Stay in your room—that is home quarantine.’ But some factories helped with that. The government has a site for quarantine [centralised government quarantine centre], right? The migrant worker can be sent there, right? After quarantine, my friend called me and told me 'Oh, we have a quarantine now, it is the government place'. ‘It is good there’, he said. MW-005 (translated from the Malay language)

#### Undocumented migrants avoid quarantine at government-designated centres for fear of arrest

Individuals testing positive for COVID-19 were mandated to quarantine at government quarantine facilities or to be hospitalised based on severity. Participants informed that undocumented migrants often evaded authorities during screening and escaped from quarantine centres to avoid immigration detention after the quarantine period.

Actually, there were quite a lot of cases [of detention after quarantine]. They [healthcare workers] would have to report you [to immigration] right? We heard of quite a few cases, where people were basically taken to [immigration] detention [centres] from the [COVID-19 quarantine] centres because of lack [of] documentation. There were quite a few people who just tried to avoid going to these [COVID-19 quarantine] centres. And some even ran away from them. So there definitely was a lot of fear. NGO-003

Undocumented migrants were reluctant to quarantine at government facilities but were unable to manage home quarantine. Participants informed that NGOs struggled to find alternate quarantine spaces, such as hotel rooms, churches and mosques, for undocumented migrants reluctant to use government facilities.

#### Revocation of free quarantine for non-citizens at government-designated centre

In January 2021, Act 342 was amended to end free quarantine for migrant workers, shifting the responsibility to employers for the payment of costs of COVID-19 investigations, treatment and admission costs to government low-risk quarantine centres. This policy-maker suggested that the policy change aimed to alleviate the government’s high expenses in maintaining quarantine centres and managing COVID-19 cases.

I think because the cost of maintaining the quarantine centres and healthcare was so phenomenal. You know, that was the reason [for the policy on making employers responsible for paying quarantine fees for migrant workers]. But in an ideal situation, you wouldn't [do that], right? Because that’s how you control a pandemic, by getting people isolated and tested and treated. It was not an easy decision, I would say, at the time. And also, I think, trying to hold employers responsible for their staff [was] right. There could be another way you look at it. POL-002

The revocation of free quarantine for non-citizens was cited as a significant factor in documented migrants’ reluctance to undergo quarantine in government facilities. Despite policy assigning quarantine costs to employers, participants noted that these expenses were prohibitive and discouraged compliance with quarantine measures.

Undocumented migrant workers and refugees were reluctant to quarantine at government facilities as they lacked employers to rely on for payment. While this policy was aimed at migrant workers, participants indicated that refugees were also impacted due to inconsistent implementation. Activists interviewed asserted that despite the policy stipulating the employer’s responsibility for healthcare expenses, interpretation varied based on officers’ understanding on the ground. If employers were unavailable, migrants were required to pay.

#### Delayed treatment may explain increased COVID-19 deaths among migrants

During the pandemic, all moderate to severe COVID-19 cases were directed to designated government hospitals equipped to manage such patients, with admission and treatment fees waived. Private hospitals did not manage COVID-19 patients.

Participants highlighted that fear of immigration enforcement at government healthcare facilities hindered migrants from seeking testing and treatment, particularly undocumented migrants, who would avoid hospitalisation. Institutional mistrust in government healthcare was identified as the primary reason for this delay. Participants further noted that migrants would wait until they were severely ill before seeking medical treatment, often resulting in poor outcomes or even death.

They would not go to the hospital unless they really felt very sick. And so that’s why even the deaths of the refugees [could be explained because] they went to the hospital at the very last minute and so their condition was quite poor and [they may have had] many other health conditions [co-morbidities] that they had made them became worse, leading to their death. So I think generally migrants will not go to the hospital unless they think it is life and death or something. NGO-005

Participants expressed concerns about underreported deaths among non-citizens, suspecting that many might have passed away at home without seeking hospital care. However, other interviewees doubted this scenario, noting that most would have notified health authorities to collect bodies to avoid potential transmission of infectious diseases.

## Discussion

Our findings suggest that the intense livelihood crisis motivated migrants to prioritise immediate survival over mitigating COVID-19 risks. Job precarity following containment measures disrupted access to food and shelter and hindered compliance with personal protection measures.^438^,^40^ Similar challenges were observed among low-income individuals in different settings, particularly those engaged in informal work without financial support, and those residing in substandard housing lacking space to social distance, or comply with stay-at-home orders.^41^,^44^ Non-citizens should receive equitable occupational and social protection, including financial assistance akin to citizens, particularly during health crises.^45 46^ Financial insecurity worsens vulnerabilities and may impede public health efforts, particularly during infectious disease outbreaks where infections spread regardless of citizenship status. Exclusionary public health measures could indirectly impact citizens, undermining prompt and efficient management of pandemics.

This study suggests that migrants primarily adhered to public health measures to avoid fines, often lacking full comprehension due to official health communication being delivered in inaccessible languages and mediums. NGOs and international organisations filled the gap by translating and disseminating COVID-19 information to migrant communities.^38^ However, official health information should be accessible, understandable and timely, with inclusive communication addressing diverse nationalities, ethnicities, languages, cultures and capabilities.^46^,^48^ Thailand exemplifies this approach with official COVID-19 helplines and infographics offered in multiple languages, as a result of national-level multistakeholder coordination involving the Ministry of Public Health, international organisations, academia and NGOs. Migrant health workers (MHWs) and migrant health volunteers (MHVs) played crucial roles in disseminating health information and fostering connections between health professionals and migrant communities, due to their proximity to the community.^49^ Involving community members in health communication strategies is a savvy localised approach that could be easily scaled up during a crisis.^50^ While community engagement in public health emergencies is widely employed in low-income countries,^51 52^ it is also relevant in high-income countries.^53 54^ In Austria, civil society organisations and social facilities contributed to enhancing the acceptance of public health measures among vulnerable individuals despite challenges faced by direct contact restrictions, prompting the need for digital communication.^55^

Employers’ failure to provide habitable housing increased COVID-19 risk for migrant workers in Malaysia, as challenges practising physical distancing, quarantine and isolation persist. Despite legislation to ensure minimum housing standards for workers, delayed enforcement and insufficient manpower for inspection hinder improvements.^3856^,^58^ Similar outbreaks in Singapore due to overcrowded housing and poorly enforced housing standards in foreign workers’ dormitories, prompted policy reforms including expanding legal mandates and constructing more purpose-built dormitories.^59 60^ Malaysia should similarly prioritise improving workers’ housing conditions to mitigate health risks associated with overcrowding, inadequate sanitation and ventilation, learning from costly lessons of the pandemic.

Our findings highlight intensified pre-existing barriers to healthcare access during the pandemic, as containment measures impacted migrants’ job stability, potentially leading to undocumented status and vulnerability to immigration enforcement and income loss. Despite the Malaysian government’s attempts at a comprehensive pandemic response, policies lacked sensitivity to marginalised communities’ needs.^24 61^ Inconsistencies between ministries, including increased immigration raids despite assurances of migrants’ exemption from arrests while healthcare-seeking, deepened mistrust in government health services.^62^,^64^ Perceived securitisation of health discouraged undocumented migrants, especially, from seeking COVID-19-related testing, quarantine, medical care and vaccinations.^65 66^ Ambiguity regarding healthcare costs for non-citizens and employers’ responsibility for testing and quarantine expenses may have delayed timely care seeking, despite free COVID-19-related healthcare in Malaysia.^67^ Globally, countries extended healthcare access during the pandemic to all residents, viewing it as a sound investment in integration, social cohesion and public health,^68^,^70^ yet practical challenges such as communication barriers and fears of deportation due to xenophobic immigration policies and racism, may have hampered implementation.^71^,^74^ We recommend widespread dissemination of information regarding healthcare services availability and access options for migrants while advocating for healthcare to be decoupled from immigration status and ability to pay.

The migrant community in Malaysia is diverse, encompassing different languages and cultural backgrounds, resulting in distinct experiences and vulnerabilities. Therefore, interviews were conducted with stakeholders, including migrants from various communities, facilitating triangulation of findings. While the findings presented here generally apply to different migrant groups in Malaysia during the pandemic, the qualitative nature of this study precludes immediate generalisation to other settings. Additionally, the vaccination programme was excluded from the reviewed public health measures in this paper due to its extensive nature, warranting further detailed examination.

## Conclusion

By shedding light on public health measures for marginalised populations during the unprecedented COVID-19 pandemic, this case study underscores the health and socioeconomic disparities faced by migrant populations in Malaysia and emphasises the urgent need for substantial reforms to safeguard migrant health beyond pandemic settings. Policy recommendations include extending social and occupational protections, offering financial assistance, improving health communication, upgrading living conditions and ensuring healthcare access for migrants during pandemic periods.

## supplementary material

10.1136/bmjph-2024-000923 online supplemental file 1

10.1136/bmjph-2024-000923 online supplemental file 2

## Data Availability

All data relevant to the study are included in the article or uploaded as online supplemental information.
