# Supplementary material for: Challenges faced by migrant populations in complying with public health measures during the COVID-19 pandemic in Malaysia: A qualitative study
Source: BMJ Public Health. 2024 Sep 4;2(2):e000923. doi: 10.1136/bmjph-2024-000923 (PMC11816197; doi:10.1136/bmjph-2024-000923)
Supplement: online supplemental file 1 [file bmjph-2-2-s001.pdf]

Appendix 1: Definition of terms

In this paper, migrant populations refer to documented and undocumented low-income migrant workers, and refugees and asylum-seekers residing in Malaysia. International travellers, students and expatriates were excluded from this study. The International Labour Organization (ILO) defines migrant workers as persons who migrate from one country to another with the purpose of work (1). Regular or documented migrant workers are those employed in another country with the requisite legal documents, like valid passports and work permits. Irregular or undocumented migrant workers are those who enter a country in search of employment without the necessary legal documents, as well as those who have entered a country legally and have either overstayed beyond the authorised period or violated the terms of their visa (2, 3). Refugees are persons forced to flee their country of origin and are unable or unwilling to return due to a well-founded fear of persecution (4). Asylum-seekers are individuals seeking international protection, but whose refugee status has yet to be determined (5).

References

1. ILO. International Labour Conference 87- Report III (IB) Migrant Workers. Geneva: International Labour Organization; 1999.
2. Key Migration Terms [Internet]. 2018 [cited 28 August 2019]. Available from: https://www.iom.int/key-migration-terms.
3. UNESCO. Social and human sciences: International migration - glossary of migration related terms 2018 [Available from: www.unesco.org/shs/migration/glossary.
4. United Nations. Convention relating to the Status of Refugees. Geneva, Switzerland: United Nations; 1951. p. 137.
5. UNHCR. UNHCR Glossary 2021 [Available from: https://reporting.unhcr.org/glossary.
