# Supplementary material for: Challenges faced by migrant populations in complying with public health measures during the COVID-19 pandemic in Malaysia: A qualitative study
Source: BMJ Public Health. 2024 Sep 4;2(2):e000923. doi: 10.1136/bmjph-2024-000923 (PMC11816197; doi:10.1136/bmjph-2024-000923)
Supplement: online supplemental file 2 [file bmjph-2-2-s002.pdf]

Interview Guide

A. <u>Documented and undocumented migrants (migrant workers, refugees, asylum seekers)</u>

Interview topics and questions that form the broad framework of discussion with migrants will include:

Topics:

- Knowledge, experience and perception of COVID-19 movement restrictions in Malaysia.
- Knowledge, experience and perception of financial difficulties and support during the COVID-19 pandemic in Malaysia.
- Knowledge, experience and perception of COVID-19 non-pharmaceutical interventions (social distancing and individual preventive measures) in Malaysia.
- Knowledge, experience and perception of COVID-19 testing and treatment in Malaysia.
- Knowledge, experience and perception of the COVID-19 vaccination programme in Malaysia.
- Experience of mental health during the COVID-19 pandemic in Malaysia.
- Experience of perceived discrimination during the COVID-19 pandemic in Malaysia.
- Suggestion for the improvement of health for migrants in Malaysia.

Introductory questions:

i. Sex
ii. Year of Birth
iii. Nationality
iv. Level of education
v. Years, location, living arrangement in Malaysia
vi. Employment or source of income
vii. Documentation status

Open questions:

1. How was your (or your friends) experience with movement restrictions during the COVID-19 pandemic in Malaysia?
   <u>Prompts</u>: Source of information on movement restrictions? Travelling to work? Travelling to buy food? Return to home country? What do you do outside of work? Compliance and acceptance?

2. How were your (or your friends) finances affected during the COVID-19 pandemic in Malaysia?
   <u>Prompts</u>: Job loss? Wages/Income? Food? Housing?

3. What support did you (or your friends) receive during the COVID-19 pandemic in Malaysia?
   <u>Prompts</u>: Source of information? Financial support? Non-financial support? Support from employer/NGO/government/local people/other migrants/others

4. How was your experience with COVID-19 individual preventive measures in Malaysia? (masking, hand washing social distancing etc)
   <u>Prompts</u>: Source of information? Understanding? Compliance and acceptance? Barriers? Information source: employer/NGO/government/local people/other migrants/others

5. How was your (or your friends) experience with access (or attempt to access) to COVID-19 testing in Malaysia?
   Prompts: Source of information? Understanding? Barriers to seeking medical care? (financial, geographic, documents etc) Support?

6. Were you (or your friends) infected with COVID-19 in Malaysia?
   Prompts: Experience in testing (workplace/home/symptomatic testing)? Experience with COVID-19 symptoms?

7. How was your (or your friends) experience with quarantine or hospitalization following infection of COVID-19 in Malaysia?
   Prompts: Source of information? Understanding? Compliance and acceptance? Barriers to seeking medical care? (financial, geographic, documents etc) Support?

8. How was your (or your friends) experience with (or attempt to access with) COVID-19 vaccination in Malaysia?
   Prompts: Source of information? Understanding? Compliance and acceptance? Barriers (financial, geographic, documents etc)? Support?

9. How did you (or your friends) feel during the COVID-19 pandemic in Malaysia?
   Prompt: Who or where did you get support from (talk to people, seek professional help, etc) when feeling down during the COVID-19 pandemic?

10. Have you ever experienced discrimination during the COVID-19 pandemic in Malaysia?
    Prompt: Could you share? Was the experience worse, the same or better than before the COVID-19 pandemic?

11. What are your suggestions for the improvement of health for migrants in Malaysia?

Interview Guide

B. **<u>Key Stakeholders: Migrant Representatives, NGOs, Civil Society, Trade Unions, Academia, Government Officials and etc.</u>**

Interview topics and questions that form the broad framework of discussion with key stakeholders will include:

Topics:

- Knowledge, experience and perception of COVID-19 movement restrictions among migrants in Malaysia.
- Knowledge, experience and perception of financial difficulties and supports among migrants during the COVID-19 pandemic in Malaysia.
- Knowledge, experience and perception of COVID-19 non-pharmaceutical interventions (social distancing and individual preventive measures) among migrants in Malaysia.
- Knowledge, experience and perception of COVID-19 testing and treatment among migrants in Malaysia.
- Knowledge, experience and perception of COVID-19 vaccination programme among migrants in Malaysia.
- Knowledge, experience and perception of mental health of migrants during the COVID-19 pandemic in Malaysia.
- Knowledge, experience and perception of perceived discrimination among migrants during the COVID-19 pandemic in Malaysia.
- Suggestion for the improvement of health for migrants in Malaysia.

Introductory questions:

<u>For representatives of migrants' communities, NGOs, Civil Society and Trade Unions:</u>

i. What is your role in your community/organisation?
ii. What communities or nationalities does your organisation represent?
iii. What is the demographic profile of migrants in your community? (age, sex, occupation, marital status)
iv. What is immigration status of the communities or organisation that you represents? (documented/undocumented, economic migrants, refugees, stateless people)
v. What is your community or organisation's role during COVID-19 pandemic for migrants? (activities, success, challenges)

Open questions:

1. Could you please share how migrants dealt with COVID-19 movement restrictions in Malaysia?
   <u>Prompt:</u> Soure of information on movement restrictions? Travelling to work? Travelling to buy food? Return to home country? What did they do outside of work? Compliance and acceptance?

2. Could you please share how migrants' finances were affected during the COVID-19 pandemic in Malaysia?
   <u>Prompt:</u> Job loss? Wages/Income? Food? Housing? Access to medical care?

3. Could you please share how migrants have been supported during the COVID-19 pandemic in Malaysia?
   Prompts: Source of information? Financial support? Non-financial support? Support from employer/NGO/government/local people/other migrants/others

4. Could you please share how migrants deal with COVID-19 individual preventive measures in Malaysia? (masking, hand washing social distancing etc)
   Prompts: Source of information? Understanding? Compliance and acceptance? Barriers? Information source: employer/NGO/government/local people/other migrants/others

5. Could you please share the experience of migrants with access (or attempt to access) to COVID-19 testing in Malaysia?
   Prompts: Source of information? Understanding? Barriers in seeking medical care? (financial, geographic, documents etc) Support?

6. Have the migrant in your community or organisation infected with COVID-19 in Malaysia?
   Prompts: Experience in testing (workplace/home/symptomatic testing)? Experience with COVID-19 symptoms? What did you or the community/organisation you represents do?

7. Could you please share how was the experience of migrants with quarantine or hospitalization following infection of COVID-19 in Malaysia?
   Prompts: Source of information? Understanding? Compliance and acceptance? Barriers in seeking medical care? (financial, geographic, documents etc) Support?

8. Could you please share the experience of migrants with (or attempt to access with) COVID-19 vaccination in Malaysia?
   Prompts: Source of information? Understanding? Compliance and acceptance? Barriers (financial, geographic, documents etc)? Support?

9. Could you please how the migrants in your community/organization feel during the COVID-19 pandemic in Malaysia?
   Prompt: Who or where did they get support from (talk to people, seek professional help, and etc) when feeling down during the COVID-19 pandemic?

10. Could you please share the experience of migrants on discrimination during the COVID-19 pandemic in Malaysia?
    Prompt: Was the experience worse, the same or better than before COVID-19?

11. What are your suggestions for the improvement of health for migrants in Malaysia?

Interview Guide

C. **Healthcare Workers**

Interview topics and questions that form the broad framework of discussion with healthcare workers will include:

Topics:

- Knowledge, experience and perception of COVID-19 non-pharmaceutical interventions (social distancing and individual preventive measures) among migrants in Malaysia.
- Knowledge, experience and perception of COVID-19 testing and treatment among migrants in Malaysia.
- Knowledge, experience and perception of COVID-19 vaccination programme among migrants in Malaysia.
- Knowledge, experience and perception of mental health of migrants during the COVID-19 pandemic in Malaysia.
- Knowledge, experience and perception of perceived discrimination among migrants during the COVID-19 pandemic in Malaysia.
- Suggestion for improvement of health for migrants in Malaysia.

Introductory questions:

i. In what capacity do you deal with migrants?
ii. What are the demographic characteristics of migrants you see? (sex, occupation, country of origin, documentation status)

Open questions:

1. From your experience, how migrants deal with COVID-19 individual preventive measures in Malaysia? (masking, hand washing social distancing etc)
   Prompts: Source of information? Understanding? Compliance and acceptance (barriers)? Information source: employer/NGO/government/local people/other migrants/others

2. Could you please share the experience of migrants with access (or attempt to access) to COVID-19 testing in Malaysia?
   Prompts: Source of information? Understanding? Barriers in seeking medical care? (financial, geographic, documents etc) Support?

3. Have you encountered migrants who were infected with COVID-19 in Malaysia?
   Prompts: Experience in testing (workplace/home/symptomatic testing)? Experience with COVID-19 symptoms?

4. From your experience, how migrants deal with quarantine or hospitalization following infection of COVID-19 in Malaysia?
   Prompts: Source of information? Understanding? Compliance and acceptance? Barriers in seeking medical care? (financial, geographic, documents etc) Support?

5. Could you please share the experience of migrants with (or attempt to access with) COVID-19 vaccination in Malaysia?
   Prompts: Source of information? Understanding? Compliance and acceptance? Barriers (financial, geographic, documents etc)? Support?

6. From your experience, how was the mental health of migrants during the COVID-19 pandemic in Malaysia?
   Prompt: What are the common issues?

7. From your experience, how the experience of migrants with discrimination while accessing (or attempt to access) health services during the COVID-19 pandemic in Malaysia?
   Prompt: Was the expereince worse, the same or better than before COVID-19?

8. Do you have difficulty communicating with migrants? How do you overcome this?

9. Do you have any suggestions for the improvement of health for migrants in Malaysia?
